# Depression in ankylosing spondylitis and the role of disease-related and contextual factors: a cross-sectional study

**DOI:** 10.1186/s13075-019-1995-7

**Published:** 2019-10-21

**Authors:** Casper Webers, Laura Vanhoof, Carsten Leue, Annelies Boonen, Sebastian Köhler

**Affiliations:** 1 Department of Internal Medicine, Division of Rheumatology, Maastricht University Medical Center, PO Box 5800, 6202 AZ Maastricht, the Netherlands; 2 Care and Public Health Research Institute (CAPHRI), Maastricht University, Maastricht, the Netherlands; 3 Department of Psychiatry and Psychology, Maastricht University Medical Center, Maastricht, the Netherlands; 4 School for Mental Health and Neuroscience (MHeNS), Maastricht University, Maastricht, the Netherlands

**Keywords:** Ankylosing spondylitis, Depression, Contextual factors, Structural equation modelling

## Abstract

**Background:**

Patients with ankylosing spondylitis (AS) have a higher prevalence of depression compared to the general population. Comorbid depression in AS likely has a multifactorial origin. While several disease-related and contextual factors have been associated with depressive symptoms in AS, a comprehensive model of their interrelations is currently lacking. Such a model could help understand the mechanisms leading to, or maintaining, depression in AS. The objectives of the current study were to determine which factors are associated with depressive symptoms in AS and to understand their underlying relationships.

**Methods:**

Data from a cross-sectional survey-based multicentre study were used. Potential determinants included both contextual and disease-related factors. Depressive symptoms were assessed by the Hospital Anxiety and Depression Subscale (HADS-D). Direct and indirect associations between risk factors and the latent depressive symptom outcome were explored using structural equation modelling. A final model was selected based on model fit criteria and clinical plausibility.

**Results:**

Among 245 patients, median HADS-D score was 3 (interquartile range 1–6), and 44 patients (18%) had a HADS-D score ≥ 8, indicating possible depression. In the final model, contextual factors significantly associated with depressive symptoms were male gender, being employed, lower income, lower mastery and worse satisfaction with social role participation. Bath AS Disease Activity Index (BASDAI) was the only disease-related factor that was associated with depressive symptoms, acted only indirectly via mastery, and its standardized total effect on depressive symptoms was smaller than that of several contextual factors. Mastery had a central role in the path diagram and mediated the effects of BASDAI, income and satisfaction with social role participation on depressive symptoms. The final model explained 64% of the variance in the depression outcome.

**Conclusions:**

Both contextual and disease-related factors are associated with depressive symptoms in AS. Mastery, the extent to which one feels in control over life and disease, has a key role in this process. Results support a relevance of self-efficacy in disease management and patient education. In order to improve patients’ mental health, research is warranted whether mastery and its relation with depression can be modified.

**Supplementary information:**

**Supplementary information** accompanies this paper at 10.1186/s13075-019-1995-7.

## Background

Ankylosing spondylitis (AS) is a rheumatic disorder leading to significant impairment in functioning and health [1]. Several studies have shown that mental health is affected in AS, including an increased risk of depression and anxiety compared to the general population [2, 3]. Next to its impact on patients’ well-being, comorbid depression in AS and other inflammatory rheumatic diseases has been linked to higher rates of hospital admissions and work disability, leading to further economic burden [4, 5]. Prevention, timely diagnosis and appropriate management of depression in AS therefore deserve attention in clinical practice. In line with this, a recent European League Against Rheumatism (EULAR) recommendation proposed screening for depression, among other comorbidities, in patients with chronic inflammatory rheumatic diseases [6].

Depression in AS likely has a multifactorial origin, with several non-mutually exclusive pathways [2]. For instance, living with the symptoms and consequences of AS, such as impaired physical functioning, pain and fatigue, could induce a depression. On the other hand, depression might also be a direct consequence of disease activity and inflammation. Research has shown that depressed individuals have higher plasma levels of C-reactive protein (CRP) and tumour necrosis factor alpha compared to non-depressed individuals [7], which has contributed to the so-called inflammatory/cytokine hypothesis of depression suggesting that inflammation contributes to the pathophysiology of depression [8].

Studies investigating factors associated with depression in axial spondyloarthritis (axSpA), or AS in particular, have generally focused on disease-related factors. Factors commonly associated with depressive symptoms in axSpA are higher disease activity (Bath Ankylosing Spondylitis Disease Activity Index [BASDAI] or AS Disease Activity Score [ASDAS]) [9–16], worse physical function (Bath Ankylosing Spondylitis Functional Index [BASFI]) [10–13, 15, 17, 18], (widespread) pain [10, 13] and worse overall health state [18]. Contextual factors, such as education, employment and coping skills, have received less attention, but female gender [15, 19], a lower level of education [12, 13], lower income [15] and lower self-efficacy [13] have been reported to be related to depression. In AS, a model of both disease-related and contextual determinants contributing to depression, and their interrelationships, is lacking. Unravelling these intricate relationships between determinants remains challenging. For this purpose, some studies investigating depression in other chronic diseases used structural equation modelling, which allows testing complex relations between both disease-related and contextual factors and thus providing insight into underlying pathways. These studies have shown that generic risk factors, and not disease-specific factors, are the major contributors of depression in these diseases [20, 21]. In AS, such a model could help understand the mechanisms leading to, or maintaining, depression and help identify patients with AS that are at an increased risk for depression and potentially require additional or different treatment.

Therefore, the objective of the current study was to explore factors associated with depressive symptoms in AS in order to develop a comprehensive model of their interrelations, including direct and indirect (i.e. mediated) effects using structural equation modelling. We hypothesized that, compared to disease-related factors, contextual factors would contribute more to depressive symptoms in AS.

## Patients and methods

### Study population

Data from the Social Participation in AS Study (SPASS) were used for this analysis. SPASS is a survey-based and cross-sectional multicentre study including patients with AS from six hospitals in the Netherlands [22]. Patients with a diagnosis of AS in 2011 were eligible if they were at least 18 years old and fulfilled the modified New York criteria for AS according to the treating rheumatologist [23]. Exclusion criteria were insufficient ability to read/understand Dutch, no internet access and the presence of life-threatening comorbidities. The study was approved by the ethics committee of the Maastricht University Medical Center, and all participants provided informed consent.

### Assessment of generic, contextual and disease-related factors

Participants completed an online survey with questions on their socio-demographic context, including the level of educational attainment (four categories), being employed (yes/no), income (four categories), work disability (yes/no) and being in a relationship (yes/no). Lifestyle was assessed by questions on smoking (yes/no), alcohol use (yes/no) and body mass index (BMI). The Short-Form 36 (SF36) was used to capture generic health state across eight domains and summarized into a Physical Component Summary (SF36-PCS) and Mental Component Summary (SF36-MCS) [24]. Personal contextual assessments included satisfaction with performance across six social roles as determined using the shortened version of Social Role Participation Questionnaire (SRPQ), with a range of 1 (worst) to 5 (best) [25, 26], and the 7-item Pearlin’s mastery scale to measure the extent to which individuals perceived themselves in control of stressors that significantly impact their lives, with a score range of 7 (lowest level of perceived control) to 28 (highest level of perceived control) [27]. Disease-specific assessments included disease duration and medication use; presence and impact of comorbidities as assessed with the modified Self-Administered Comorbidity Questionnaire (SCQ) [28]; presence of extra-articular manifestations (EAMs; psoriasis, uveitis and inflammatory bowel disease); scores on the BASDAI and BASFI to assess disease activity and physical function, respectively [29, 30]; and spinal pain during the past week as measured using a numerical rating scale (0–10).

### Assessment of depressive symptoms

The outcome of the current study, severity of depressive symptoms, was assessed using the depression subscale of the Hospital Anxiety and Depression Scale (HADS-D) [31]. The HADS-D contains seven items, each scored on a Likert scale ranging from 0 to 3 (answering options differ per question), resulting in a range of 0 (no depressive symptoms) to 21 (high depressive symptoms). A HADS-D score ≥ 8 indicates possible depression (see Additional file 1 for the HADS-D questionnaire).

### Statistical analysis

We worked towards our final model in a pre-specified stepwise manner. Independent variables were first tested in univariable analyses. For this, negative binomial regression was used as HADS-D scores showed a positive skew and overdispersion. Variables used were socio-demographic context (age, gender, education, income, being employed, being in a relationship), lifestyle (BMI, current smoking, current alcohol use), history of depression, somatic comorbidity, generic physical health (SF-36PCS, vitality domain of SF-36), disease-specific health characteristics (disease duration, spinal pain, BASDAI, BASFI, EAMs) and finally the personal contextual factors satisfaction with social role participation and mastery. Education and income were converted into dichotomous variables (higher education/university versus other levels, annual income > €40,000 versus ≤ €40,000, respectively). Variables were considered for further analysis if they were associated at an alpha level of *p* < 0.20 with HADS-D in univariable analyses. We chose to be most inclusive at this stage before selecting out variables while working towards the parsimonious model.

To develop an explanatory model, structural equation modelling (SEM) was used. The objective was to set up a model that explained a substantial part of the variance in the outcome and had clinical plausibility, while being as parsimonious as possible (reflected by both number of included parameters and model fit indices). The SEM has a measurement and a structural part. For the measurement part, we regressed the seven manifest (observed) items of the HADS-D questionnaire on a continuous latent (unobserved) variable, which was called ‘depression’ and used to reflect depressive symptoms. The advantage of this method, compared to simply using the observed HADS-D score, is that only the individual items’ *shared* variance contributes to the factor ‘depression’. In contrast, each item’s *unique* variance is considered nuisance/measurement error and does not contribute to the factor ‘depression’. As a result, the depression construct is represented more accurately by this factor ‘depression’. We used a weighted least squares means and variance-adjusted estimator as the individual HADS items were measured on an ordinal Likert scale.

The structural component consisted of direct and indirect paths between variables which were thought to be associated with depression. Direct paths go directly from a variable to depression, while indirect paths go from a variable via another variable (or multiple variables) to depression. We tested two approaches: a *theory-driven* and a *data-driven* approach. First, a pure *theoretical model* was specified based on existing evidence and clinical plausibility. Then, its model fit was compared to a simple ‘baseline’ model with only direct paths to depression. In subsequent data-driven steps, paths in this baseline model that did not contribute substantially (*p* < 0.10) were eliminated one by one using manual backward selection. Subsequently, possible improvement of the model by specifying indirect paths between variables was explored based on modification indices (MIs) and substantive interpretation of results, finally leading to a *data-driven model*. Only indirect paths that were deemed to be clinically plausible were explored. Models were evaluated by absolute and incremental fit indices, notably the root mean square error of approximation (RMSEA; value < 0.05 indicates good fit), comparative fit index (CFI; value ≥ 0.95 indicating good fit) and Tucker-Lewis fit index (TLI; value ≥ 0.95 indicating good fit) [32]. The latter two are incremental fit indices and can also be used to compare fit across different models. Statistical analyses were performed with Stata Release 14 (StataCorp LP, USA) and Mplus version 8 (Muthén & Muthén, USA).

## Results

Table 1 shows the characteristics of the study population. In total, 246 patients completed the SPASS surveys. HADS-D scores were missing for one patient. In the remaining 245 patients, the mean HADS-D score was 4.1 (median = 3, interquartile range = 1–6). Forty-four patients (18%) had an increased HADS-D score (≥ 8) indicating possible depression.

**Table 1:** Characteristics of the study population

Variable	Total (*n* = 245)*
Age, years	51.2 (12.3)
Male gender, *n* (%)	153 (62.4)
High education, *n* (%)^†^	80 (32.7)
Currently employed, *n* (%)	139 (56.7)
High income, *n* (%)‡	95 (38.8)
SRPQ (satisfaction, 1–5)	3.2 (0.8)
Mastery (7–28)	20.7 (3.9)
Comorbidity (SCQ)	3.1 (4.3)
History of depression, *n* (%)	9 (3.7)
Disease duration, years	23.6 (13.3)
History of any EAM, *n* (%)	78 (31.8)
History of psoriasis, *n* (%)	20 (8.2)
History of IBD, *n* (%)	33 (13.5)
History of uveitis, *n* (%)	44 (18.0)
NSAID use, *n* (%)	134 (54.7)
Biological use, *n* (%)	125 (51.0)
BASDAI (0–10)	4.4 (2.3)
BASFI (0–10)	4.2 (2.6)
Patient global (0–10)	4.8 (2.7)
SF36-PCS (0–100)	38.8 (10.8)
SF36-MCS (0–100)	49.2 (12.8)
HADS-depression (0–21)	4.1 (3.7)

Values are expressed as mean (SD) unless otherwise stated

*BASDAI* Bath Ankylosing Spondylitis Disease Activity Index, *BASFI* Bath Ankylosing Spondylitis Functional Index, *EAM* extra-articular manifestation, *HADS* Hospital Anxiety and Depression Scale, *IBD* inflammatory bowel disease, *NSAID* nonsteroidal anti-inflammatory drug, *SCQ* Self-Administered Comorbidity Questionnaire, *SF36-PCS* Short-Form 36 Physical Component Summary, *SF36-MCS* Short-Form 36 Mental Component Summary, *SRPQ* Social Role Participation Questionnaire

*One out of 246 patients did not complete the HADS questionnaire and is therefore excluded

†Higher education/university

‡Annual income > €40,000

### Identification of candidate variables for SEM

In univariable analyses, male gender, being employed, lower income, not being in a relationship, no current alcohol use, higher BMI, comorbidity, history of depression, history of EAMs, longer disease duration, higher BASDAI, higher BASFI, more back pain, lower SF-36PCS, lower SF36 vitality domain score, lower mastery and less satisfaction with role performance (SRPQ) were associated with increased depressive symptoms at *p* < 0.20 (Additional file 2). Hence, these variables were retained in the next modelling step.

### SEM of the theoretical model

**Fig. 1:**
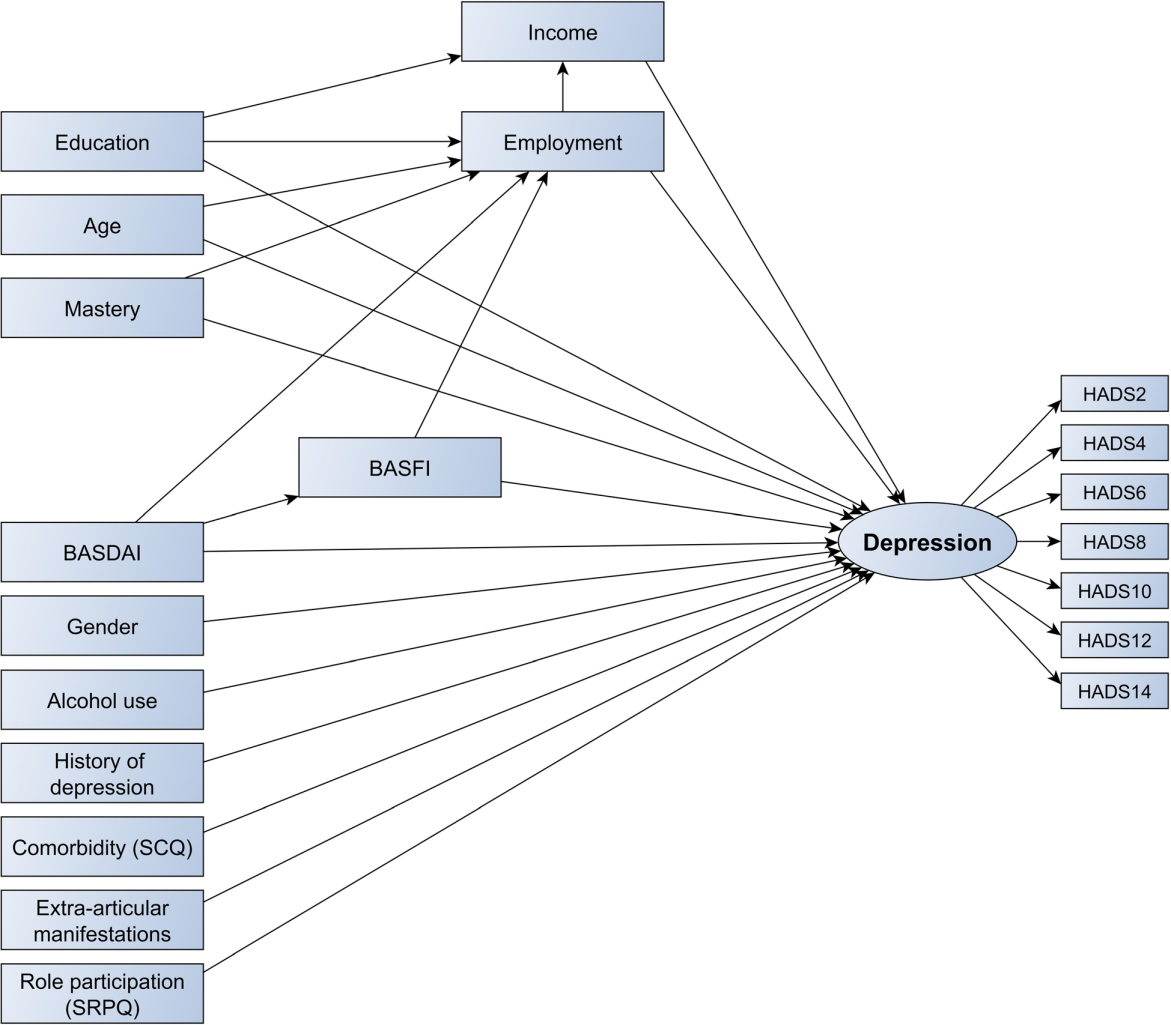
Initial (theoretical) model of depression in ankylosing spondylitis. This model was the initial, hypothesized model of depression in AS, based on biological plausibility and existing evidence. Standardized regression coefficients are presented in Table 2. The numbered HADS items represent the individual corresponding items on the HADS questionnaire. BASDAI, Bath Ankylosing Spondylitis Disease Activity Index; BASFI, Bath Ankylosing Spondylitis Functional Index; HADS, Hospital Anxiety and Depression Scale; SCQ, Self-Administered Comorbidity Questionnaire; SRPQ, Social Role Participation Questionnaire

The theoretical model is shown in Fig. 1 and further specified in Table 2. Next to the factors identified above, we included age and education based on existing evidence. As we preferred disease-specific outcome measures over generic outcome measures, SF-36 PCS (correlated with BASFI), SF-36 vitality domain and spinal pain (both correlated with BASDAI) were excluded due to collinearity. In addition, disease duration (correlated with age) and relationship status (correlated with SRPQ) were excluded. Age was preferred over disease duration, as the latter was self-reported and might be inaccurate. SRPQ was preferred over being in a relationship as the former was considered to capture a broader range of social roles. Contextual factors finally retained comprised age, gender, education, income > €40,000, employment, alcohol use, history of depression, SRPQ and mastery. Disease-related factors were BASDAI, BASFI, comorbidity and presence of any EAM. The theory-driven model had insufficient fit to the data (RMSEA = 0.058, CFI = 0.878, TFI = 0.849). In comparison, the baseline model including only direct paths between independent variables and the depression outcome showed moderate fit (RMSEA = 0.041, CFI = 0.932, TFI = 0.918). Therefore, the theoretical model was rejected.

**Table 2:** Standardized estimates of associations between variables, theoretical model

Variable	Dependent	*B*	SE	*p*
Age	Depression	0.023	0.064	0.715
Male gender	Depression	0.186	0.057	0.001
Education, high*	Depression	0.090	0.061	0.143
Income, high†	Depression	− 0.088	0.070	0.206
Employed, yes	Depression	0.178	0.073	0.014
Alcohol use, yes	Depression	0.015	0.059	0.803
Mastery (7–28)	Depression	− 0.495	0.070	< 0.001
SRPQ (1–5)	Depression	− 0.351	0.072	< 0.001
History of depression	Depression	0.060	0.066	0.361
Comorbidity score (SCQ, 0–39)	Depression	0.015	0.068	0.830
BASDAI (0–10)	Depression	0.052	0.079	0.510
BASFI (0–10)	Depression	0.023	0.063	0.716
EAM, any	Depression	− 0.033	0.058	0.571
Age	Employed, yes	− 0.530	0.061	< 0.001
Education, high	Employed, yes	0.031	0.086	0.717
Mastery (7–28)	Employed, yes	0.115	0.114	0.314
BASDAI (0–10)	Employed, yes	0.152	0.114	0.185
BASFI (0–10)	Employed, yes	− 0.210	0.092	0.022
BASDAI (0–10)	BASFI (0–10)	0.676	0.041	< 0.001
Education, high	Income, high	0.410	0.068	< 0.001
Employed, yes	Income, high	0.131	0.098	0.181

Standardized regression coefficients can be interpreted as the change in *y* (dependent variable) in *y* standard deviation units for a standard deviation change in *x* (independent variable)

*BASDAI* Bath Ankylosing Spondylitis Disease Activity Index, *BASFI* Bath Ankylosing Spondylitis Functional Index, *EAM* extra-articular manifestation, *SCQ* Self-Administered Comorbidity Questionnaire, *SE* standard error, *SRPQ* Social Role Participation Questionnaire

*Higher education/university

^†^Annual income > €40,000

### SEM of the data-driven model

**Fig. 2:**
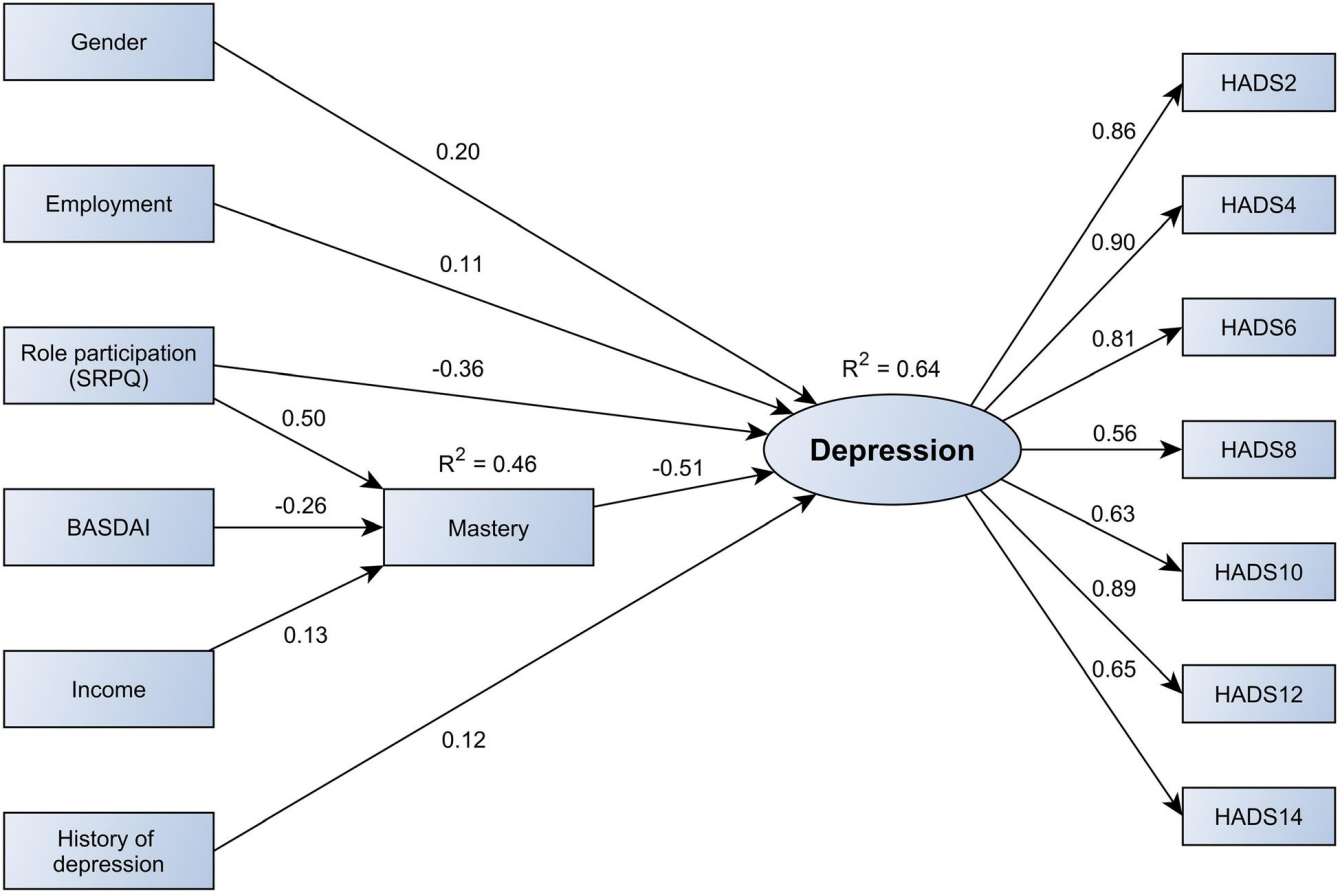
Final model of depression in ankylosing spondylitis. This model is the final model of depression in AS, based on model fit criteria. Numbers above the arrows represent the standardized regression coefficients of direct paths. Standardized regression coefficients can be interpreted as the change in *y* (dependent variable) in *y* standard deviation units for a standard deviation change in *x* (independent variable). Standardized coefficients of indirect effects are presented in Table 4. The numbered HADS items represent the individual corresponding items on the HADS questionnaire. BASDAI, Bath Ankylosing Spondylitis Disease Activity Index; HADS, Hospital Anxiety and Depression Scale; SRPQ, Social Role Participation Questionnaire

The baseline model (model 1) as mentioned above was then subject to further specification. First, non-significant paths were removed in a backward fashion (age, alcohol use, BASFI, presence of comorbidities, EAM). This model had good fit (model 2; RMSEA = 0.034, CFI = 0.970, TFI = 0.963). Then, indirect paths between contextual factors (education, employment, income, satisfaction with social role participation and mastery) and disease-related factors (such as BASDAI) were explored step-by-step to see whether this would improve the explanatory power of the model further. To avoid capitalization on spurious associations in the data, paths were foremost added based on clinical plausibility. During this modelling step, the non-significant path from education to depression was dropped, and indirect paths from BASDAI, income and social role participation to depression via mastery were added. This model (model 3) had good absolute fit according to RMSEA (0.038) and better incremental fit according to CFI (0.980) and TFI (0.975) than model 2. After careful consideration, and in light of model fit, explained variance in the outcome and model parsimony, model 3 was considered the final model. The model is presented in Fig. 2 and specified in Tables 3 and 4.

**Table 3:** Standardized estimates of associations between variables, final model

Variable	Dependent	*B*	SE	*p*
Male gender	Depression	0.197	0.061	0.001
Employed, yes	Depression	0.107	0.060	0.076
SRPQ (1–5)	Depression	− 0.363	0.066	< 0.001
Mastery (7–28)	Depression	− 0.507	0.064	< 0.001
History of depression, yes	Depression	0.121	0.072	0.092
SRPQ (1–5)	Mastery	0.495	0.044	< 0.001
BASDAI (0–10)	Mastery	− 0.262	0.060	< 0.001
Income, high*	Mastery	0.133	0.059	0.025

Standardized regression coefficients can be interpreted as the change in *y* (dependent variable) in *y* standard deviation units for a standard deviation change in *x* (independent variable)

*BASDAI* Bath Ankylosing Spondylitis Disease Activity Index, *SE* standard error, *SRPQ* Social Role Participation Questionnaire

*Annual income > €40,000

**Table 4:** Standardized direct and indirect effects of mediated paths on depression, final model

Variable	Direct/indirect	*B*	SE	*p*
SRPQ (1–5)	Direct	− 0.363	0.066	< 0.001
	Indirect (via mastery)	− 0.251	0.037	< 0.001
	Total	− 0.614	0.053	< 0.001
BASDAI (0–10)	Direct	N/A		
	Indirect (via mastery)	0.133	0.037	< 0.001
	Total	0.133	0.037	< 0.001
Income, high*	Direct	N/A		
	Indirect (via mastery)	− 0.067	0.032	0.033
	Total	− 0.067	0.032	0.033

Standardized regression coefficients can be interpreted as the change in *y* (dependent variable) in *y* standard deviation units for a standard deviation change in *x* (independent variable)

*BASDAI* Bath Ankylosing Spondylitis Disease Activity Index, *N/A* not applicable, *SE* standard error, *SRPQ* Social Role Participation Questionnaire

*Annual income > €40,000

### Contextual and disease-related factors in the final model

Model 3 explained 64.2% of the variance in the latent depression outcome. Male gender, history of depression, being employed and decreased mastery were directly associated with increased depressive symptoms. In addition, higher BASDAI, lower satisfaction with role participation and an annual income ≤ €40,000 were indirectly associated with increased depressive symptoms (all via mastery, Table 4). Notably, BASDAI was the only disease-related factor in the model. Its standardized total effect on depression (*B* = 0.133) was smaller than that of SRPQ (*B* = − 0.614) and male gender (*B* = 0.197), comparable to that of a history of depression (*B* = 0.121) and being employed (*B* = 0.107) and greater than that of income (*B* = − 0.067) (Tables 3 and 4).

### Sensitivity analyses

Several post hoc sensitivity analyses were conducted to test the robustness of results. First, the association between being employed and depressive symptoms could have been confounded by age. To exclude a possible effect of age, the final model was applied to the population aged 65 or younger (65 was the legal age of retirement in the Netherlands in 2011). This led to similar results: being employed was still associated with more depressive symptoms (*p* = 0.03). Second, history of depression was dropped from the final model (as we could not exclude that those with a previous diagnosis of depression never recovered and were still in the same depressive episode). The associations between other determinants and depressive symptoms remained similar, but model fit worsened. Third, we investigated whether BASFI could (a) substitute BASDAI, (b) should be included next to BASDAI or (c) mediated the association between BASDAI and mastery. All scenarios resulted in lower model fit, lower explained proportion of the variance in the outcome or non-significant paths from BASFI to depression (*p* = 0.30).

## Discussion

In this cross-sectional study of a representative cohort of Dutch AS patients, both contextual and disease-related factors were related to depressive symptoms in AS and together explained a substantial proportion of its variance. Higher mastery, a personal contextual factor, was particularly associated with lower depressive symptoms and an important mediator for several other factors. Regarding disease-related factors, BASDAI was the only determinant in the final explanatory model. Its association with depression was mediated by mastery and weaker than that of several contextual factors.

Our study suggests that mastery plays a central role for the presence of depressive symptoms in AS. A higher sense of mastery, or perceived control over things that happen to an individual in life, has been associated with better employment status in AS and with better mental and physical well-being in other chronic diseases [33–35]. In line with our results, the related construct of lower self-efficacy has previously been associated with depression in AS [13, 36]. Importantly, our data show that mastery not only has a direct relation with depression, but also is a strong mediator for other factors as a sort of final common (psychological) pathway. More specifically, higher disease activity, being unable to satisfactorily perform social roles and lower income are associated with decreased feelings of control (mastery), which in turn is associated with increased depressive symptoms. These findings could have important implications for the prevention and treatment of depressive symptoms by increasing patients’ sense of mastery. The central role of mastery supports the focus on increasing self-management skills of patients with AS. Available education programmes for patients with inflammatory arthritis nowadays not only focus on transferring knowledge, but also aim to improve cognitive and behavioural coping [37]. Such interventions might affect directly mastery, which is considered to be a main determinant or precedent of coping, but they might also help patients with lower levels of mastery cope with specific stressors associated with being chronically ill, without affecting mastery as a personality trait.

In contrast, disease-related factors (BASDAI) had a more modest association and only indirectly so through mastery. This is in line with previous research in a general population sample showing that mastery mediates the relationship between functional limitations and depressive symptoms [38]. Unfortunately, we did not have data on direct measures of inflammation, e.g. CRP or erythrocyte sedimentation rate (ESR), leaving uncertainty on a direct role of inflammation in patients with auto-inflammatory disease, independent of their experience of stiffness and fatigue. The relationship between inflammation and depression is complex [8]. While increased self-reported disease activity (possibly a proxy of inflammation) has consistently been associated with depressive symptoms in several studies, results vary for the relation between CRP/ESR and depressive symptoms [39]. Of note, sensitivity analyses showed BASDAI likely is a better determinant of depressive symptoms than BASFI. Possibly, BASDAI reflects inflammation more appropriately (in line with the inflammation hypothesis) [40]. Alternatively, the symptoms as reflected by the BASDAI (pain, stiffness, fatigue) might have a greater impact on the affective state than functional impairments. Finally, it could be that the effect of functional impairments was already represented by other variables in the model, such as being employed or role participation.

Besides mastery, three other contextual factors, namely male gender, employment and a history of depression, were directly associated with depressive symptoms. While it is known that female patients with AS report worse quality of life, male patients have higher inflammatory markers [41], suggesting our findings are consistent with the neuro-inflammatory hypothesis of depression [8, 42]. Nonetheless, increased depressive symptoms in male patients are in contrast with studies in the general population [43]. Moreover, a study investigating the risk of depression in patients with AS and controls found no substantial interaction between AS and gender [3], while another recent study did find no association between gender and depressive symptoms in multivariable analysis [15]. Obviously, confirmation of gender differences in depression from clinical studies of AS is needed, including insight into gender-specific pathways in depression [44].

Being employed is generally considered beneficial for physical and mental health [45]. Surprisingly, we found that being employed was associated with increased depressive symptoms, even after adjusting for satisfaction with work performance as captured by the SRPQ [22]. It should be noted, however, that the SRPQ assesses satisfaction with role participation, which is subject to personal interpretation and does not necessarily reflect objective difficulties with role performance. One could speculate that, despite a (potential) positive effect of employment on well-being, employment may also lead to role overload, role conflict and role strain in patients with chronic disease [46].

A past history of depression has been shown to be an important risk factor in both the general population and those with chronic disease [20, 21, 47]. Previous episodes of depression likely mark an underlying vulnerability. However, few patients reported a history of depression (4%), which might reflect under-detection of affective states in patients with complex somatic and psychiatric comorbidities [48]. Also, it is unknown whether patients who reported a history of depression had recovered from this, or had an ongoing depression at the time that the survey was conducted.

The study has some notable strengths. From an etiological perspective, development of depression should be considered a multifaceted process, in which factors can have both direct and indirect effects [47]. SEM allowed us to investigate the dependencies between both observed variables and the latent construct by using a path model. Other strengths were the participation of six centres within the Netherlands with different settings (academic and private hospitals) and from various geographical regions, which increases generalizability, and the availability of data on multiple contextual factors.

The current study also has some limitations. First, although the HADS-D is a validated instrument to assess depressive symptoms, it is a self-reported screening tool [49]. Abnormal HADS-D scores do not necessarily correspond to a diagnosis of major depressive disorder and should not be considered as such. Of note, the HADS-D does compare favourably to other measures of depressive symptoms with regard to predictive value [49]. Second, due to the cross-sectional design of this study, no firm conclusions regarding causality can be drawn. Although the good fit of the final model supports the relationships between variables as specified in our model, these need to be replicated in a longitudinal study. Third, the mean disease duration of patients in SPASS was high, limiting the generalizability to patients with recent onset of disease.

Almost one fifth of the patients in this cohort had a HADS-D score that indicates ‘possible depression’. In other studies, these rates are even higher [39]. Apparently, depressive symptoms (and likely depression) are common in AS, which supports screening for depressive symptoms in practice [6]. Based on the current study, it cannot be stated whether mastery can be improved (nor whether an improvement in mastery would lead to improvement of psychological health). However, patients with low mastery might be at increased risk of depression in AS. As such, screening for low mastery might be helpful to identify those who need additional support. In addition, patient education could be tailored to the individual patient’s context, including their level of mastery.

## Conclusions

In summary, the current study showed that both contextual and disease-related factors are associated with depressive symptoms in AS. Mastery, the extent to which one feels in control over life and disease, likely has a key role in this process.

Timely diagnosis and management of depression in AS will improve patients’ health and likely save societal costs. Future studies should investigate how mastery can be enhanced, and whether this results in better mental well-being in AS.

## Supplementary information

**Additional file 1.** Items of the depression subscale of the Hospital Anxiety and Depression Scale (HADS-D). Contains the individual items of the depression subscale of the Hospital Anxiety and Depression Scale, including the scoring method.

**Additional file 2.** Univariable negative binomial regression of HADS-depression scores in patients with ankylosing spondylitis. Table with the results of the univariable negative binomial regression analysis of possible determinants of depressive symptoms in ankylosing spondylitis.

## Data Availability

All data generated or analysed during this study are included in this published article and its supplementary information files.

## Abbreviations

AS: Ankylosing spondylitis
ASDAS: Ankylosing Spondylitis Disease Activity Score
axSpA: Axial spondyloarthritis
BASDAI: Bath Ankylosing Spondylitis Disease Activity Index
BASFI: Bath Ankylosing Spondylitis Functional Index
BMI: Body mass index
CFI: Comparative fit index
CRP: C-reactive protein
EAM: Extra-articular manifestation
ESR: Erythrocyte sedimentation rate
EULAR: European League Against Rheumatism
HADS-D: Depression subscale of the Hospital Anxiety and Depression Scale
MI: Modification index
RMSEA: Root mean square error of approximation
SCQ: Self-Administered Comorbidity Questionnaire
SEM: Structural equation modelling
SF36: Short-Form 36
SF36-PCS: Short-Form 36 Physical Component Summary
SF36-MCS: Short-Form 36 Mental Component Summary
SPASS: Social Participation in Ankylosing Spondylitis Study
SRPQ: Social Role Participation Questionnaire
TLI: Tucker-Lewis fit index
