# Supplementary material for: Depression in ankylosing spondylitis and the role of disease-related and contextual factors: a cross-sectional study
Source: Arthritis Res Ther. 2019 Oct 21;21:215. doi: 10.1186/s13075-019-1995-7 (PMC6805406; doi:10.1186/s13075-019-1995-7)
Supplement: Supplementary file 1 — Additional file 1. Items of the depression subscale of the Hospital Anxiety and Depression Scale (HADS-D). Contains the individual items of the depression subscale of the Hospital Anxiety and Depression Scale, including the scoring method. [file 13075_2019_1995_MOESM1_ESM.docx]

**Additional File 1. Items of the depression subscale of the Hospital Anxiety and Depression Scale (HADS-D) (**[**1**](#_ENREF_1)**)**

Note: patients are asked to choose the reply that is closest to how they have been feeling in the past week.

HADS2. I still enjoy the things I used to enjoy:

| 0 | Definitely as much |
| --- | --- |
| 1 | Not quite as much |
| 2 | Only a little |
| 3 | Hardly at all |

HADS4. I can laugh and see the funny side of things:

| 0 | As much as I always could |
| --- | --- |
| 1 | Not quite so much now |
| 2 | Definitely not so much now |
| 3 | Not at all |

HADS6. I feel cheerful:

| 3 | Not at all |
| --- | --- |
| 2 | Not often |
| 1 | Sometimes |
| 0 | Most of the time |

HADS8. I feel as if I am slowed down:

| 3 | Nearly all the time |
| --- | --- |
| 2 | Very often |
| 1 | Sometimes |
| 0 | Not at all |

HADS10. I have lost interest in my appearance:

| 3 | Definitely |
| --- | --- |
| 2 | I don’t take as much care as I should |
| 1 | I may not take quite as much care |
| 0 | I take just as much care as ever |

HADS12. I look forward with enjoyment to things:

| 0 | As much as I ever did |
| --- | --- |
| 1 | Rather less than I used to |
| 2 | Definitely less than I used to |
| 3 | Hardly at all |

HADS14. I can enjoy a good book or radio or TV program:

| 0 | Often |
| --- | --- |
| 1 | Sometimes |
| 2 | Not often |
| 3 | Very seldom |

**Scoring**

Individual items are added, resulting in a sum score with range 0 (best) to 21 (worst).

**References**

1. Zigmond AS, Snaith RP. The hospital anxiety and depression scale. Acta Psychiatr Scand. 1983;67(6):361-70.
