# Supplementary material for: Depression in ankylosing spondylitis and the role of disease-related and contextual factors: a cross-sectional study
Source: Arthritis Res Ther. 2019 Oct 21;21:215. doi: 10.1186/s13075-019-1995-7 (PMC6805406; doi:10.1186/s13075-019-1995-7)
Supplement: Supplementary file 2 — Additional file 2. Univariable negative binomial regression of HADS-depression scores in patients with ankylosing spondylitis. Table with the results of the univariable negative binomial regression analysis of possible determinants of depressive symptoms in ankylosing spondylitis. [file 13075_2019_1995_MOESM2_ESM.docx]

**Additional File 2. Univariable negative binomial regression results of HADS-depression scores in patients with ankylosing spondylitis**

| **Variable** | **IRR** | **95% CI** | **p** |
| --- | --- | --- | --- |
| Age, years | 1.00 | 0.99-1.01 | 0.88 |
| Gender, male | 1.27 | 1.01-1.60 | 0.04 |
| Education, high* | 0.90 | 0.71-1.14 | 0.38 |
| Employed, yes | 0.79 | 0.63-0.98 | 0.03 |
| Income, high† | 0.74 | 0.59-0.93 | 0.01 |
| Relationship, yes | 0.76 | 0.57-1.00 | 0.05 |
| Smoking, current | 1.01 | 0.76-1.34 | 0.95 |
| Alcohol use, yes | 0.81 | 0.63-1.06 | 0.13 |
| BMI | 1.02 | 1.00-1.05 | 0.06 |
| Mastery (7-28) | 0.86 | 0.84-0.88 | <0.01 |
| SRPQ (1-5) | 0.50 | 0.45-0.57 | <0.01 |
| Comorbidity (SCQ) (0-39) | 1.04 | 1.01-1.06 | <0.01 |
| History of depression, yes | 2.07 | 1.22-3.52 | <0.01 |
| History of psoriasis, yes | 0.88 | 0.58-1.32 | 0.53 |
| History of uveitis, yes | 1.20 | 0.90-1.59 | 0.21 |
| History of IBD, yes | 1.43 | 1.05-1.95 | 0.02 |
| History of any EAM, yes | 1.21 | 0.96-1.53 | 0.11 |
| Disease duration, years | 1.01 | 1.00-1.01 | 0.16 |
| Biological use, yes | 1.31 | 1.02-1.58 | 0.02 |
| NSAID use, yes | 0.94 | 0.75-1.17 | 0.58 |
| BASDAI (0-10) | 1.14 | 1.08-1.20 | <0.01 |
| BASFI (0-10) | 1.14 | 1.09-1.19 | <0.01 |
| Back pain NRS (0-10) | 1.16 | 1.12-1.21 | <0.01 |
| SF36-PCS (0-100) | 0.98 | 0.97-0.99 | <0.01 |
| SF36 vitality domain | 0.95 | 0.94-0.96 | <0.01 |

*Higher education/university

†Annual income >€40,000
BASDAI, Bath Ankylosing Spondylitis Disease Activity Index; BASFI, Bath Ankylosing Spondylitis Functional Index; BMI, Body Mass Index; EAM, extra-articular manifestation; HADS, Hospital Anxiety and Depression Scale; IBD, inflammatory bowel disease; IRR, incidence risk ratio; NSAID, nonsteroidal anti-inflammatory drug; SCQ, self-administered comorbidity questionnaire; SF36-PCS, Short-Form 36 Physical Component Summary; SF36, Short-Form 36; SRPQ, Social Role Participation Questionnaire; 95% CI, 95% confidence interval.
